# A novel Bruton’s tyrosine kinase gene (*BTK*) missense mutation in a Chinese family with X-linked agammaglobulinemia

**DOI:** 10.1186/1471-2431-14-265

**Published:** 2014-10-15

**Authors:** Bixia Zheng, Yayuan Zhang, Yu Jin, Haiguo Yu

**Affiliations:** Department of Gastroenterology, Nanjing Children’s Hospital Affiliated to Nanjing Medical University, Nanjing, 210008 China; Department of Rheumatology and Immunology, Nanjing Children’s Hospital Affiliated to Nanjing Medical University, Nanjing, 210008 China

**Keywords:** X-linked agammaglobulinemia, Bruton tyrosine kinase, Germline mutation

## Abstract

**Background:**

X-linked agammaglobulinemia (XLA) is a rare inherited disease characterized by recurrent bacterial infections, a paucity or absence of peripheral lymphoid tissue, an absence of circulating B cells, and marked depression of serum IgG, IgA, and IgM. Germline mutation of the *BTK* gene has been identified as a cause of XLA. These mutations cause defects in early B cell development.

**Case presentation:**

In this study, we report a variant form of XLA with partial B cell function that results from a missense mutation (c.1117C > G) in exon 13 of the *BTK* gene. A genetic analysis of the family revealed an affected male sibling with a c.1117C > G mutation. He was observed with low level of serum immunoglobulin and CD19+ B cell and received the IVIG replacement therapy regularly in follow up. Four female carriers were found.

**Conclusion:**

*BTK* mutation analysis is necessary in the diagnosis of XLA and may be used for subsequent genetic counseling, carrier detection and prenatal diagnosis.

## Background

X-linked agammaglobulinemia (XLA), also known as Bruton agammaglobulinemia, is the major immunodeficiency disease (PID) identified in childhood [1]. XLA is a primary immunodeficiency disease caused by a severe block of B cell development at the pre-B to immature B cell stage that is induced by mutations of the human Bruton’s tyrosine kinase (*BTK*) gene [2]. The main clinical symptoms of XLA include recurrent severe infections and dramatically decreased B cell and immunoglobulin levels [3].

The *BTK* gene is located at Xq21.3-Xq22 and encompasses 37.5 kb that contain 19 exons. The *BTK* protein that is encoded by the *BTK* gene has 5 different functional domains; i.e., the PH, TH, SH3, SH2, and TK domains [4]. Any mutation that occurs in any site within one of these 5 domains can affect the activity of the tyrosine kinase and thus influence the maturation of pre-B cells [5, 6]. Here, we reported a case of XLA that was induced by a *BTK* gene mutation and the results of examinations of the genetic mutations in the patient’s family.

## Case presentation

A 6-year-old boy was admitted to our hospital for recurrent intermittent fever for more than 2 years. This recurrent fever reached a peak temperature of 39°C and was initially found in June of 2011. His temperature decreased to within the normal range following after anti-infective therapies. In the subsequent 2 years, the boy experienced 1 episode of septicemia (*staphylococcus aureus*), 3 episodes of otitis media (etiology unknown), 2 episodes of pneumonia (*staphylococcus aureus* and *streptococcus pneumoniae*) and 4 episodes of bronchitis (etiology unknown). Anti-infective therapies were administered for these episodes to restore normal temperatures. This boy again exhibited cough and fever one month prior to admission to our hospital for further diagnosis and treatment. Upon admission, X-ray revealed patchy high-density shadows that were distributed along the lower left lobe of the lungs and mild *bilateral pulmonary emphysema.* Sputum culture revealed *streptococcus pneumoniae* that was sensitive to ceftriaxone. The patient’s temperature returned to normal after a 1-week treatment with ceftriaxone. No hepatosplenomegaly or lymphadenopathy was identified despite the recurrent infections of the patient. A routine blood examination was also performed, and the results were as follows: WBC 11.77 × 10^9 /L, N 29.8%, L 60.1%, Hb 119 g/L, PLT 304 × 10^9/L, CRP 47 mg/L, and ESR 23 mm/H. Blood biochemistry examination revealed the following: Alanine aminotransferase (ALT)5 U/L, Aspartate aminotransferase (AST )24 U/L, Lactate dehydrogenase (LDH) 320 U/L, Creatine kinase (CK) 72 U/L, Creatine kinase-MB CK-MB 23 U/L, Alpha-hydroxybutyrate dehydrogenase( HBDH )275 U/L, Total protein (TP )57.3 g/L, Albumin 40.6 g/L, and Globulin 16.7 g/L. The renal function and electrolyte levels of the patient were normal. The ferritin level was 287.4 ng/ml (normal, 80–130 ng/ml). The immune parameters were consistent with primary agammaglobulinemia:IgG < 0.34 g/L (normal, 6–12 g/L), IgA <0.264 g/L (normal, 0.7-3 g/L), IgM 0.179 g/L (normal, 0.5-3 g/L), and the level of circulating CD19 + B-lymphocytes was dramatically reduced to 0.2%, (normal 5-15%, absolute count: 25 /mm^3^). XLA was diagnosed based on the combination of profound hypogammaglobulinemia of all three immunoglobulin isotypes, the low CD19+ B-lymphocyte count together and the male gender. The final diagnosis was confirmed by molecular DNA analysis.

### Genetic analysis

**Figure 1:**
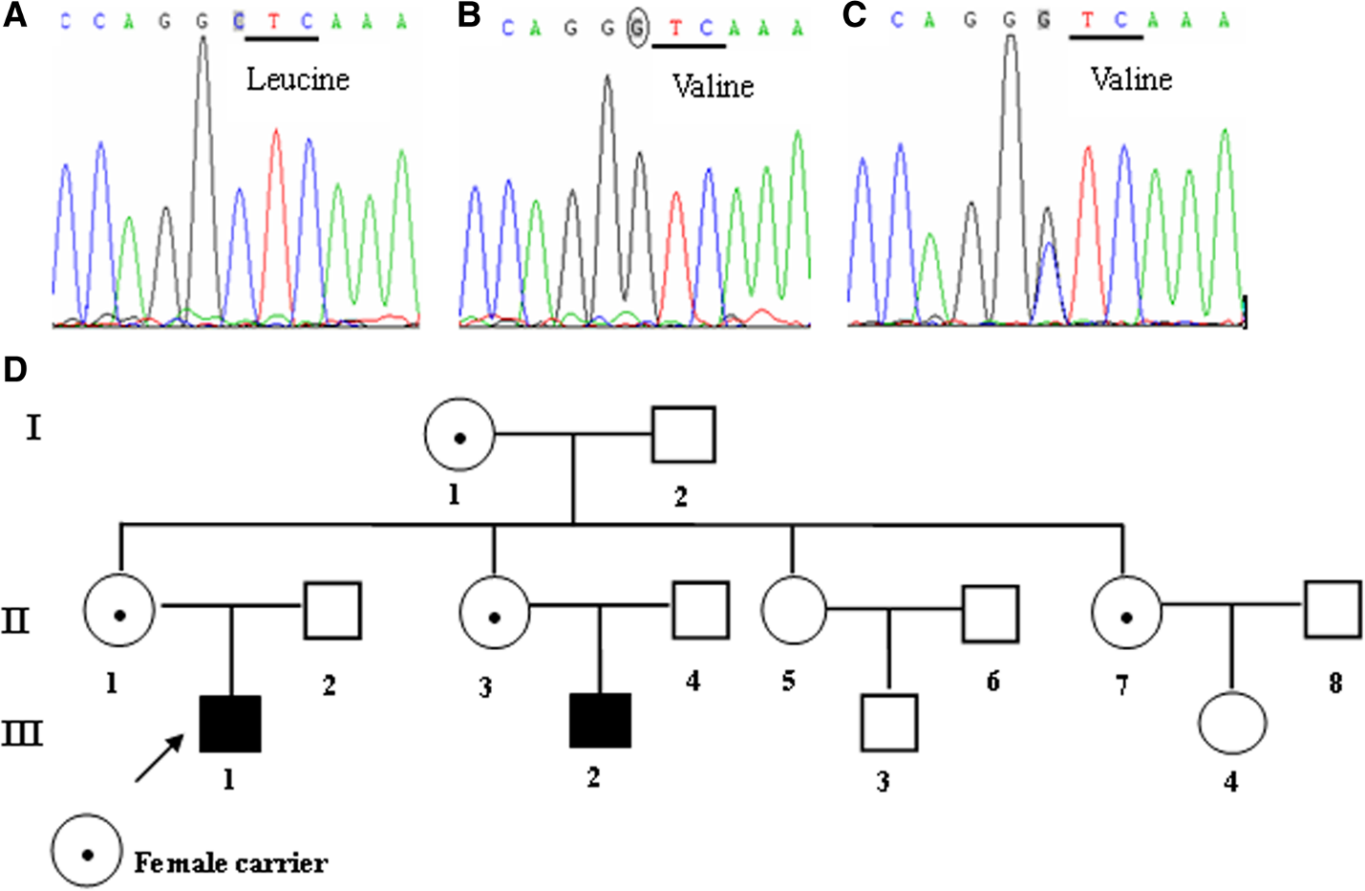
**Identification of the BTK gene mutation. (A)** The segment of exon 13 of BTK in the normal family members. **(B)** Mutation in the segment of exon 13 in the BTK in the hemizygous mutation proband. **(C)** The segment of exon13 of BTK in the heterozygous family members. **(D)** Heredity map of the family with the proband (III-1).

After informed consent had been obtained, genomic DNA was extracted from peripheral blood samples for molecular genetic analysis of the *BTK* gene. Sequencing of the *BTK* coding regions revealed a point mutation, c.1117C > G, that resulted in the amino acid substitution L373V in the SH2 domain (Figure 1B). To our knowledge, this is the first report of the mutation c.1117C > G at exon 13 in the literature.

After the proband diagnosis was confirmed, the patient’s 13 living family members were informed, and consent was obtained from these family members for purpose of genetic analysis. Genetic analysis of the family members revealed an affected 1-year –old male cousin with a c.1117C > G mutation (III-2). We observed significant CD19+ B cell deficiency (1.0%,absolute count: 55/mm^3^) with very low serum IgG 3.05 g/L, IgA 0.02 g/L, IgM 0.08 g/L. He did not present a severe infections with the exception of mild upper respiratory infections. Four female family members had a heterozygous c.1117C > G mutation (II-1, II-3, II-7, and I-1) (Figure 1C). The other members were normal and free of any genetic mutations in *BTK*.

### Analysis of the family pedigree

The analysis of the family pedigree of the proband (Figure 1D) after completing all the findings of DNA sequencing, clinical and other laboratory studies indicated that the proband in the family had an inherited X-linked recessive manner, as described in the literature by other authors [7].

### Prediction of functional consequence of the c.1117C > G mutation

The novel missense mutation c.1117C > G was shown to be conserved in different species (Table 1). Computational predictive analysis was undertaken using two algorithms: SIFT, PolyPhen. These were tested to determine their accuracy in predicting whether missense variants would be deleterious to function. c.1117C > G is predicted to be deleterious with a score of 0.00(DAMAGING) for SIFT, 0.996 (PROBABLY DAMAGING) for PolyPhen. These evidences documented that the novel mutation identified in this study is disease-causing.

**Table 1:** **Comparative alignment of the amino acid sequence of BTK**

Species	Gene	AA	Alignment
Human	ENST00000308731	373	H Q H N S A G L I S R **L** K Y P V S Q Q N K N A
Mutated	-	373	H Q H N S A G L I S R **V** K Y P V S Q Q N K N A
Ptroglodytes	ENSPTRG00000022104	373	H Q H N S A G L I S R **L** K Y P V S Q Q N K N A
Mmulatta	ENSMMUG00000019573	373	H Q H N S A G L I S R **L** K Y P V S Q Q N K N A
Mmusculus	ENSMUSG00000031264	373	H Q H N S A G L I S R **L** K Y P V S K Q N K N A
Ggallus	ENSGALG00000004958	371	H Q H N S A G L I S R **L** K Y P V S R H Q K S A
Trubripes	ENSTRUG00000012144	356	H Q H N A A G M V S R **L** K Y I V T T R M R
Drerio	ENSDARG00000004433	352	H Q H N A A G L V S R **L** K Y I V S N R A Q N A
Fcatus	ENSFCAG00000015395	373	X X X X X X X L I S R **L** K Y P V S Q Q N K N A
Xtropicalis	ENSXETG00000018152	364	H Q H N A A G L I S R **L** K Y P V C S L R K T A

Amino acid conservation is indicated by boldface type.

## Discussion

X-linked agammaglobulinemia (XLA) is a primary immunodeficiency disease that is caused by mutations in the Bruton’s tyrosine kinase (*BTK*) gene. Symptoms, including recurrent severe bacterial infection, hypogammaglobulinemia, and a remarkable reduction or lack of B cells in the peripheral blood, can be found in most of the patients with XLA shortly after birth [8]. As an X-linked recessive disease, the incidence rate of XLA is around 0.5/100,000 [9]. Bruton’s tyrosine kinase (*BTK*) gene is acknowledged as the cause of XLA and was identified by positional cloning [10]. The *BTK* protein is a member of the non-receptor protein tyrosine kinases of Tec; these proteins can catalyze the phosphorylation of tyrosine residues on various proteins and play important roles in the signaling pathway that controls the development of B lymphocytes. Mutations in any domain of the *BTK* can induce dysfunction of the *BTK* protein, block the development of pre-B cells from naive B cells, and decrease the lifespan of mature B lymphocytes. The lack of B lymphocytes and plasmocytes in the peripheral blood can decrease the synthesis of different immunoglobulins, decrease specific responses to various antigens, and thus ultimately induce immunodeficiency [11].

The *BTK* gene is localized at Xq21.3-Xq22 and encompasses 37.5 kb that contain 19 exons. The first exon of the *BTK* gene is a non-coding region, and the other 18 exons code the *BTK* protein, which contains 659 amino acids and has a relative molecular mass of 76000 Da [12]. The most comprehensive database for BTK gene variations is the BTKbase, Version 8.53 last updated 17 June, 2013 contains 1254 public entries. To date, 787 mutations of the *BTK* gene that are associated with XLA have been found in the Human Gene Mutation Database (http://www.hgmd.cf.ac.uk/ac/gene.php?gene=BTK). Most of these mutations are missense mutations, followed by nonsense mutations, splice site mutations, insertions, and deletions. Missense mutations primarily occur in the first two bases of a triplet codon. Studies have reported that mutations in the *BTK* gene can occur in the exons, introns, and promoters [13, 14]. In the present study, we provide the first report of a novel missense mutation, c.1117C > G, that was found in exon 13 and resulted in a leucine to valine amino acid substitution (L373V) in the encoded protein. The result of analyses with 2 online softwares (PolyPhen-2 and SIFT) suggested that this mutation affected the structure and function of the protein. Sequencing analysis of exon 13 within the family pedigree revealed that two male family members had the disease due to the c.1117C > G mutation and that 4 female members were c.1117C > G carriers.

This BTK genetic study is the most useful in atypical XLA patient,whose serum IgG concentration are above the diagnostic threshold and whose resulting clinical manifestation are subtle. The treatment of XLA includes prophylactic therapy with IVIG and appropriate antibiotics for acute and chronic infections. Earlier reports suggest that high dose IVIG replacement therapy (>400 mg/kg every 3 weeks) is more effective than low-dose (<200 mg/kg) in patients with XLA [15]. In our patient, gammaglobulin replacement therapy (IVIG, 400–600 mg/kg) was administered following the diagnosis. This therapy consisted of infusions of gammaglobulin once every month for the first 3 months followed by subsequent infusions once every 2–3 months. The concentration of serum IgG was 7.6 g/L in the follow up after the first administration. The serum IgG levels were also monitored and maintained above 5 g/L. No episodes of respiratory tract infection or fever were found during a 1-year follow up. Gammaglobulin replacement therapy is non-curative for the patients with XLA. Work from laboratories demonstrates the feasibility of using gene-corrected HSCs to complement the immune defects of *BTK*-deficiency in mice [16]. We propose the clinical program of stem cell based therapy for XLA should be started in the very near future.

## Conclusions

These findings suggest that genetic analysis of the *BTK* gene and evaluation of the immune function of suspected patients could increase the diagnosis rate and help clinicians to perform IVIG replacement therapy in a timely manner, which might significantly decrease the incidence of complications and the mortality rate. Genetic analysis of the *BTK* gene might also aid the identification of other patients and carriers in the patient’s family and might be used for subsequent genetic counseling.

## Consent

Written informed consent was obtained from the patient’s parents for publication of this Case report and any accompanying images. A copy of the written consent is available for review by the Editor-in-Chief of this journal.

### Ethics

The study protocol was approved by the ethics committee of the Children’s Hospital of Nanjing Medical University.

## Abbreviations

XLA: X-Linked Agammaglobulinemia
*BTK*: Bruton tyrosine kinase
PID: Primary immunodeficiency disease
PH: Pleckstrin Homology
TH: Tec Homology
SH2: Scr Homology 2
SH3: Scr Homology 3
IVIG: Intravenous immune globulin.
